# Quality attributes of set yogurt made from lactoperoxidase system activated cow’s milk

**DOI:** 10.1016/j.heliyon.2023.e17507

**Published:** 2023-06-24

**Authors:** Kedir Awol Assen, Mestawet Taye Asfaw, Binyam Kassa Engidasew

**Affiliations:** a Department of Animal Science, College of Agriculture & Natural Resources, Werabe University, P O Box: 46, Werabe, Ethiopia; b School of Animal & Range Sciences, College of Agriculture, Hawassa University, P O Box: 05, Hawassa, Ethiopia; c ET ALIM Consulting, Addis Ababa, Ethiopia

**Keywords:** Fermentation, Microbial, Sensory quality and set yoghurt

## Abstract

The study aimed to assess the quality attributes of set yoghurt made from cow's milk preserved by lactoperoxidase system (LPs). Fresh cow's milk was collected from *Datto* milk collection center and samples were grouped to control and LPs activated. Set yoghurt was prepared immediately for the controlled samples, but in LPs activated milk, sample was kept for 9 h at 25 °C ± 2 before yoghurt was made. LP enzyme was activated within 2 h after milking by adding 1 mg/ml of sodium thiocyanate (SCN^_^) and hydrogen peroxide (H_2_O_2_). Totally, 36 L of cow's milk was used and the experiment was done in triplicates. Titratable acidity (TA) and pH were assessed every 30 min until coagulation formed. Yoghurt samples were stored for 21 days and different parameters were assessed including incubation duration, pH, TA, microbiological quality and sensory attributes. The results revealed that LPs partially suppressed the rate of acid production during incubation of cultured milk. Yoghurt made from LPs treated milk had significantly (P < 0.05) higher pH and lower TA values. Similarly, yoghurt made from LPs treated milk had lower total bacterial counts at 24-h, 72-h and 7-days of storages. Additionally, yoghurt from LPs activated milk had lower yeast and mold counts at 24-h and 7-days. Yoghurt made from LPs activated milk received better scores for the sensory attributes. In general, LPs activated milk can be used for making set yoghurt without a negative effect. Further studies are needed to illustrate the LPs activated milk fitness in making other cultured milk products.

## 1 Introduction

Major milk constituents can be preserved by the manufacturing of products such as yoghurt to have a longer shelf-life. Yoghurt is one of the most popular fermented dairy products worldwide, with great consumer acceptance due to its basic nutrition and health benefits [1]. *Streptococcus thermophilus* and *Lactobacillus bulgaricus* are the two very well-known starter microorganisms causing desirable biochemical effects involved in the development of aroma, flavor, taste, texture, and increasing the sensory quality of the product [2,3].

Demand for dairy products has grown steadily and continues to grow, particularly in the urban centers of developing countries [4]; Aspects of milk quality are receiving more attention from both society and the government [5]. Consumers are increasingly concerned about the quality of products and the production conditions [6]. Relaying on these, modern milk processing in Ethiopia has introduced products of controlled fermentation, heat treatment, and enzymatic manipulation of milk for the domestic market [7].

However, milk production in Ethiopia is highly dominated by smallholder dairy producers. This production system is not different from those in many other developing countries where milk quality is challenged by factors. Milk processors are challenged with the supply of inferior milk quality since milk producers are also challenged by a lack of cold storage facilities, a lack of transport infrastructures, and shortage of capital [8,9].

To overcome problems associated with maintaining the quality of milk, an alternative method has been developed. Lactoperoxidase system (LPs) is a chemical method for preserving raw milk where refrigeration is not available [10], allows temporary control of poor milk quality and transportation to the site of dairy processing plants [11,12,13]. The lactoperoxidase system comprises three essential components: lactoperoxidase, thiocyanate, and hydrogen peroxide. Lactoperoxidase is a natural constituent of milk [14,15], while thiocyanate ion and hydrogen peroxide are at suboptimal levels. The antimicrobial activity is quite weak and lasts for 2 h. So, exogenous activation of natural system is very necessary.

Definitely, the lactoperoxidase system is essential for temporary preservation of raw milk during collection and transportation to processing plants, especially in areas with hot climate condition [13,16]. So far, in Ethiopia, there is inadequate information on the possibility of making fermented milk products, including yoghurt, from LPs activated cow's milk. Therefore, this study was conducted to determine the effect of LPs activation of raw cow's milk on the quality as well as fermentation characteristics of set yoghurt.

### 1.1 Research hypotheses

HO1: there is no significant difference between the yoghurt made from LPs activated milk and controlled in fermentation characteristics during the initial storage periods.

HO2: there is no significant difference between the yoghurt made from LPs activated milk and controlled in Lactic acid bacterial counts.

HO3: there is no significant difference between the yoghurt made from LPs activated milk and controlled in sensory attributes during the initial storage periods.

## 2 Materials and methods

### 2.1 Milk sample sources and LPs activation procedures

Raw cow's milk was collected from Datto milk collection center, Hawassa. Totally, 36 L of milk was used (18 L of milk per group). Lactoperoxidase enzyme activation was done within 2 h by adding 14 ml of freshly prepared solution of 1 mg/ml sodium thiocyanate per liter of milk as a source of thiocyanate ion. After 1 min of thorough mixing, 10 ml of freshly prepared solution of 1 mg/ml H_2_O_2_ was added and mixed for 1 min [17]. Samples were analyzed in the Dairy Science Laboratory at Hawassa University.

### 2.2 Yogurt making procedures and study design

Groups of set yoghurt, one group from LPs treated and one group from LPs untreated, were made following normal yoghurt-producing methods [18]. Thermophilic starter cultures for Direct Vat Set (DVS) were used to produce the yoghurt according to the manufacturer's recommended dose. Briefly, milk for each type of yoghurt was heat treated at 85 °C for 5 min. Then cooled to incubation temperature (43-45 °C) and incubated with thermophilic starter culture until coagulation.

#### 2.2.1 Study design

Yoghurts made from both types of milk (LPs activated and controlled) were divided into two main groups before incubation. One of the groups consisted of samples intended for analysis during incubation. The second group consisted samples intended for analysis during storage. During incubation, pH and titratable acidity were assessed every 30 min while waiting for coagulation. The Yoghurt samples were stored at 6–8 °C and analyzed at 0, 1, 2, 3, 7, 14, and 21 days of storage. The experiment was done in triplicate.

### 2.3 Laboratory analysis

#### 2.3.1 Acidity and pH determination of yoghurt

The evaluation of LPs fermentation characteristics were monitored by measuring the titratable acidity and pH values. The pH value was measured using pH meter (Orion Research Inc., Cambridge, MA). Lactic acid production was determined by titration of 9 ml of sample and taken in a sterile conical flask, and then three drops of phenolphthalein indicator was added to make the color change with NaOH (0.1 N) to a light pink color. Finally, titratable acidity was expressed in percent lactic acid [19].

#### 2.3.2 Yoghurt microbiological composition analysis

Microbiological composition analysis was done by using fitting agars for each species of microbe (Table 1). One ml of samples were added into sterile test tubes containing nine ml of peptone water up to a serial dilution of 10-^7^ and mixed thoroughly. Appropriate decimal dilutions were selected that would give the expected number of colonies (between 30 and 300). Samples with duplication were placed on a petri-dish, and then molten agar medium (15–20 ml) was poured onto the petri-dish and mixed thoroughly.

#### 2.3.3 Sensory analysis

Acceptability tests for yoghurt samples were performed using panelists. A total of twenty five consumers took part in the sensory analysis, and they were requested to evaluate the sensory attributes of the set yoghurt samples and fill out the questionnaire prepared. Consumer panelists were selected based on the following criteria: age between 18 and 64 years, and they had to be “consumers” of fermented milk products. Bottled water was provided to the panelists to rinse their mouths after each taste. Tests took place at room temperature with equalized light intensity levels, free from disturbing noise and with a continually circulating air. The taste, color, flavor, smell, texture, and overall acceptability of yoghurt samples were evaluated by using a five-point hedonic scale, where five was given for the best sensory quality and one was given for samples with the least quality yogurt based on the panelists (5 = like very much, 4 = like moderately, 3 = neither like nor dislike, 2 = dislike moderately, and 1 = dislike very much). Yoghurt samples were presented in a random fashion. The sensory evaluation was conducted at Hawassa University dairy science laboratory [20].

### 2.4 Statistical analysis

The response variables were analyzed following the GLM procedure of SAS 9.4 (SAS Institute, USA) [21]. First, the colony forming units per milliliter (CFU/ml) were converted in to natural logarithms. PROC GLM procedure of SAS was used to determine statistically significant differences (P < 0.05). Means comparisons were done by Duncan test for variables that showed significant differences at the 5% significance level. This study applied the following model for the analysis of response variables:

Yij = μ + Li + Sj + eij; Where, Yij = the response variables; μ = overall mean; Li = Lactoperoxidase activation effect; Sj = Storage durations and eij = random error.

### 2.5 Ethical considerations

The research proposal was evaluated and approved by the Research Review Committee of the College of Agriculture at Hawassa University, and ethical clearance was obtained (HU/CA/024/27/03/12018). Verbal agreement was obtained from each of the sensory analysis participants after explaining the purpose of the study prior to the start of data collection. The respondents were informed about their voluntary participation and their full legitimate rights to withdraw at any time.

### 2.6 Strengths and limitations of the study

The current study was designed to assess the suitability of raw cow's milk preserved by lactoperoxidase system (LPs) activation for the manufacture of set yoghurt. Yoghurt storage characteristics were assessed including pH, lactic acid percentage, microbiological and sensory. However, the effects of continuous LPs induction on oxidative stress and free radical were not included in this research work.

## 3 Results and discussion

### 3.1 Fermentation characteristics of yoghurt milk during production

The results in the rate of acid development and pH changes were recorded in 30-min intervals until the desired coagulation was attained. Initially, at 0-hr, 30 min and 1-h after incubation there were comparable acidity development between LPs activated, and the control portion (Fig. 3). However, after 1:30 h of incubation, the rate of acid development was faster in the control (0.75), and it was significantly higher as compared to the LPs activated milk (0.73). Thereafter, during the remaining hours of incubation, no significant variations were observed (P > 0.05) in acidity development between the LPs activated and the control group.

Under LPs activated groups, the titratable acidity increased progressively, and there was a significant difference (*P < 0.05*) in the titratable acidity of samples among the average values (Fig. 3). Similarly, within the control yoghurt samples, rate of acid development increased progressively. There was significant difference (*P < 0.05*) in the titratable acidity of samples among the average values, except between the average values at 3:30 and 4:00 h.

**Fig. 1:**
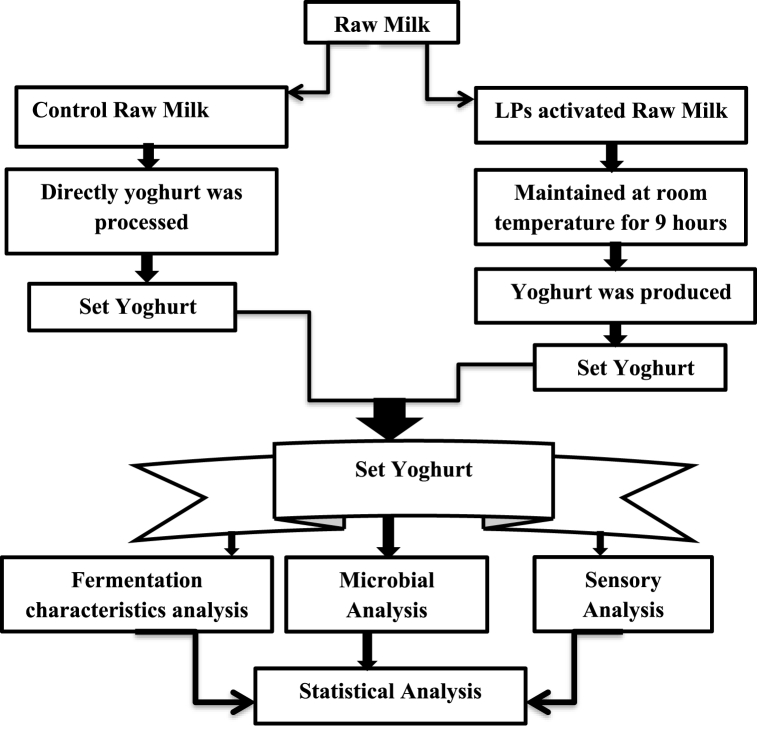
Experimental structure arrangement of yoghurt processing steps.

Lower acid development was observed in the LP system activated milk sample after 1:30 h of incubation (Fig. 3). This hints that LP system might have a retarding effect on lactic acid bacteria, resulting in slow acid development in the activated milk, as starter cultures are sensitive to antimicrobial agents from LP system components, although it did not affect the overall acid development (see Fig. 1).

**Fig. 2:**
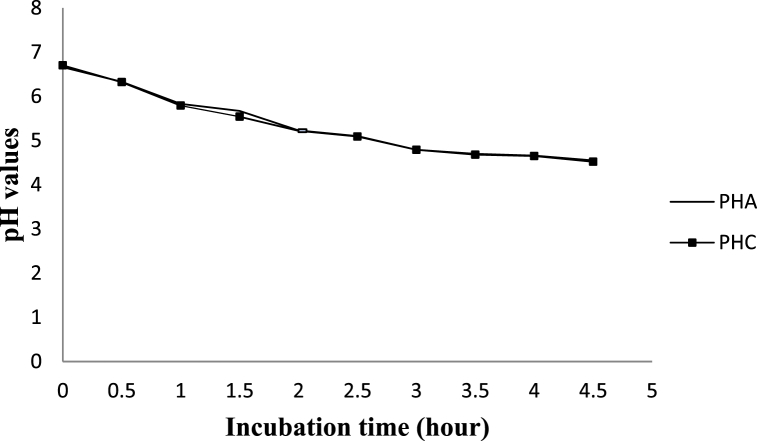
The pH values of set yoghurt from LPsystem treated and control milk samples during incubation.

**Fig. 3:**
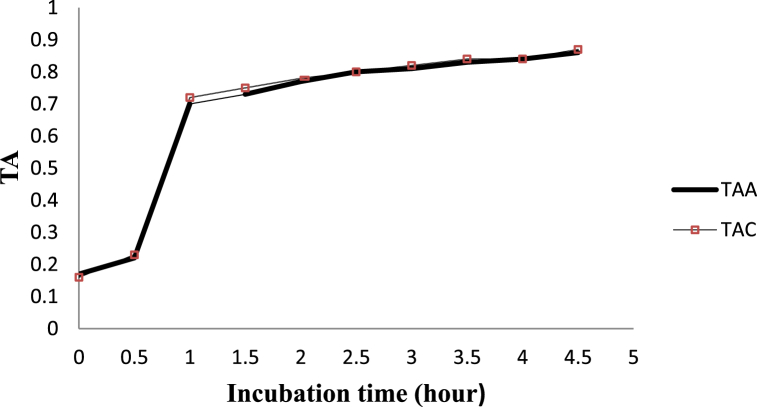
Acid Development (%lactic acid) values of set yoghurt from LPsystem treated and control milk samples during incubation. Abbreviations: pHC-pH value of control. pHA-pH value of LPs treated. TAA-titratable acidity of LPs treated, TAC- titratable acidity of control.

After 1:30 h of incubation, the pH in the control (5.54) dropped significantly faster (*P < 0.05)* as compared to the LPs-treated milk (5.67). However, at all the remaining hours of incubation, the average pH values of yoghurt from LPs treated and control milk samples were similar (P > 0.05), which might be due to the bacteriostatic effects of the system. Within LPs activated portion, the pH values decreased progressively (Fig. 2). There were significant differences (*P < 0.05*) in the pH values of samples among the mean values of incubation hours. Similarly, in the control group, pH reduced progressively. There was a significant difference (*P < 0.05*) in pH values of samples among the mean values but not at 3:00 and 4:00 h. Entirely, samples from the control milk had a higher acidity percentage (Fig. 3) and a lower pH value than those from the activated samples at some incubation points (Fig. 2). However, at the final stage of desired coagulum formation, there were no significant differences due to treatments.

The finding from the current study agrees with that of Ponce [22,9], where there were similar percentages of lactic acid and coagulation times between LPs activated and non-activated yoghurt samples. However, a study by Bekele et al. [23] reported that the acidification speed of the activated cow milk was delayed by 2-h as compared to control cow milk to reach the desired pH value. Lower acid development in the activated sample was observed during incubation, showing that the LP system has an inhibitory effect on lactic acid bacteria, which retarded acid formation in the LPs activated milk [24,16,9]. Masud et al. [25] found reduced acidity and a prolonged incubation period for yoghurt from activated milk.

### 3.2 Fermentation characteristics of yoghurt during storage

During storage, there were significant (*P < 0.05*) differences in average pH values between the yoghurt from LPs activated and control milk groups at the 3rd, 7th, 14th, and 21st days (Table 2). A significantly lower (*P < 0.05*) pH was observed in yoghurt from activated milk. With respect to titratable acidity, there were no significant differences (*P > 0.05*) between yoghurt from LPs and control groups at 0-hr, 24-hrs and 48-hrs of storage. However, significant differences (*P < 0.05*) were observed on the 3rd, 7th, 14th and 21st days of storage. Generally, yoghurt from LPs activated milk had a higher pH and lower TA values than yoghurt from control milk mainly during the last days of storage (Table 2**)**. This could be an important contribution towards maintaining the quality of yoghurt, by inhibiting sharp acidity development.

**Table 1:** Microbial growth media and incubation period.

Species of Microbes	Growth Media	Incubation hours	References
Total bacterial count	Total Plate Count Agar	32 °C for 48 h	[26]
Coliform count	Violet Red Bile Agar	32 °C for 24 h	[27]
Total staphylococcus counts	Mannitol Salt Agar	35 °C for 45–48 h	[28]
Yeast and Molds	Potato Dextrose Agar	25 °C for 3–5 days	
Lactic acid bacteria	MRS (De Mans, Rogosa and Sharpe) agar	37 °C for 48 h	[29]

**Table 2:** Acid development (% lactic acid) and pH values of set yoghurt during storage.^a^

Parameters		Group	Storage Durations (hour)						
			0	24	48	72	7-day	14-day	21-day
pH		LPs	4.55 ± 0.01^f^	4.52 ± 0.00^e^	4.47 ± 0.01^d^	4.45 ± 0.00^c^	4.44 ± 0.00^cb^	4.43 ± 0.00^b^	4.41 ± 0.00^a^
		Cont	4.52 ± 0.02^c^	4.50 ± 0.01^c^	4.45 ± 0.01^b^	4.41 ± 0.00^a^	4.40 ± 0.01^a^	4.38 ± 0.01^a^	4.38 ± 0.01^a^
P value			0.061	0.058	0.271	0.144	0.032	0.047	0.026
Acidity	LPs		0.84 ± 0.01^a^	0.86 ± 0.00^a^	0.92 ± 0.02^b^	0.94 ± 0.00^cb^	0.95 ± 0.00^cd^	0.95 ± 0.01^cd^	0.97 ± 0.00^d^
	Cont		0.86 ± 0.01^a^	0.87 ± 0.00^a^	0.94 ± 0.00^b^	0.96 ± 0.00^c^	0.96 ± 0.00^c^	0.98 ± 0.00^d^	1.00 ± 0.00^d^
P value			0.073	0.082	0.163	0.041	0.023	0.011	0.047

Abbreviations: LPs, Lactoperoxidase system; NS, non-significant; Con, control.

^a^ Data presented are the means of triplicates with standard errors. Means in the same row with different superscripts are significantly different (P < 0.05). Storage duration was mentioned in hours, if not indicated.

This result is in agreement with studies by Ndambi et al. [24,9], who noticed activation of the LP enzyme delayed lactic acid formation in yoghurt during storage. Study by Srisaikham [30] also indicated that the LPs system application is mainly used in dairy products like yoghurt to control acidity for 14 days of storage. Similarly, Kussendrager and Hooijdonk [31] found that LPs treatment of milk controls the acidity of yogurt.

### 3.3 Microbiological attributes of yoghurt

**Fig. 4:**
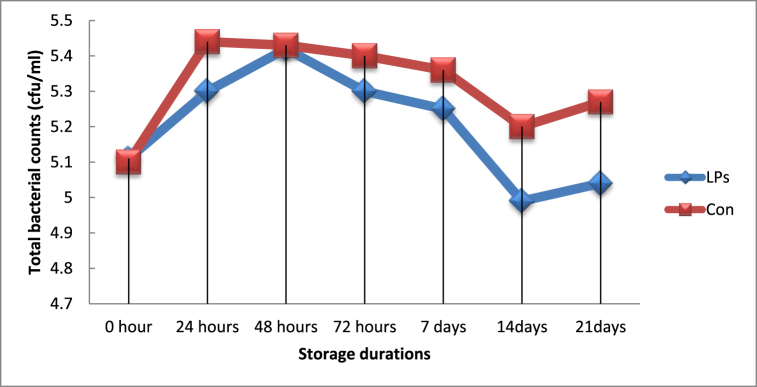
Total bacterial counts in yoghurt made from LPs activated and control milk samples.

There was a significant difference (*P < 0.05*) in total bacterial counts between yoghurt from activated and non-activated groups of milk at 24-h, 72-h, and 7-days of storage, and lower counts were recorded in yoghurt made from LPs treated milk (Fig. 4). However, during the remaining storage periods, significant differences (*P > 0.05*) were not observed, as shown in Table 3. Contrary to the current result, Magdoub et al. [32] and Nakada et al. [33] reported that the addition of LPs did not affect the bacterial growth in yoghurt samples. Regarding lactic acid bacteria count, throughout the storage period, lactic acid bacteria counts did not differ significantly (*P > 0.05*) between yoghurt from LPs activated and control milk groups. This indicates that LPs did not show bactericidal effects on the lactic acid bacteria (Fig. 6).

**Table 3:** Microbial counts in LP activated and control yoghurt samples.^a^

Para.	Gro.	Storage Duration (hours)						
		0	24	48	72	7 day	14day	21day
TBC	LPs	5.11 ± 0.06	5.30 ± 0.01	5.42 ± 0.04	5.30 ± 0.03	5.25 ± 0.01	4.99 ± 0.30	5.04 ± 0.29
	Con	5.10 ± 0.07	5.44 ± 0.03	5.43 ± 0.03	5.40 ± 0.01	5.36 ± 0.02	5.20 ± 0.28	5.27 ± 0.30
P value		0.091	0.046	0.128	0.039	0.001	0.051	0.066
LAB	LPs	5.63 ± 0.25	6.23 ± 0.27	5.80 ± 0.16	6.12 ± 0.30	6.15 ± 0.39	5.65 ± 0.52	5.38 ± 0.32
	Con	5.96 ± 0.41	6.23 ± 0.26	5.93 ± 0.21	5.98 ± 0.27	6.21 ± 0.39	5.64 ± 0.31	5.57 ± 0.29
P value		0.212	0.091	0.053	0.077	0.187	0.093	0.055
YM count	LPs	4.71 ± 0.01^a^	5.18 ± 0.02^ab^	5.27 ± 0.11^b^	5.49 ± 0.02 ^b^	5.31 ± 0.02^b^	5.10 ± 0.31^ab^	5.35 ± 0.31^b^
	Con	4.71 ± 0.02^a^	5.34 ± 0.01^b^	5.17 ± 0.13^ab^	5.55 ± 0.03^b^	5.46 ± 0.02^b^	5.36 ± 0.38^b^	5.48 ± 0.30^b^
P value		0.065	0.021	0.058	0.077	0.046	0.083	0.054

Abbreviations: LPs, Lactoperoxidase system; NS, non-significant; Con, control; TBC, total bacterial count; LAB, lactic acid bacteria; YM, yeast and mold.

^a^ Data presented are the means of triplicates with standard errors. Means in the same row with different superscripts are significantly different (P < 0.05).

**Fig. 5:**
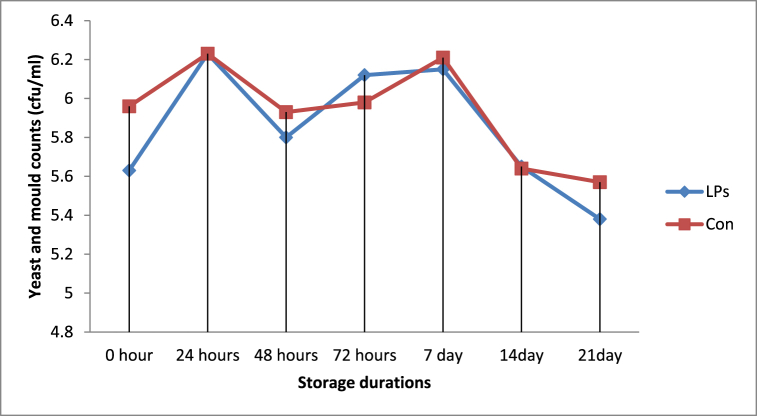
Yeast and mold counts in yoghurt made from LPs activated and control milk samples.

The yeast and mold counts at 24-hr and 7-day storage periods showed that the average counts of yeast and mold differed significantly (*P < 0.05*) between yoghurt samples from LP system activated and non-activated milk (Fig. 5). Yeast and mold counts in the yoghurt samples made from milk preserved by the LP system were lower than those in the yoghurt samples made from control milk in most of the storage periods. Under both yoghurt groups, 0-day counts were significantly lower (*p < 0.05*) as compared to the last storage period (Table 3).

**Fig. 6:**
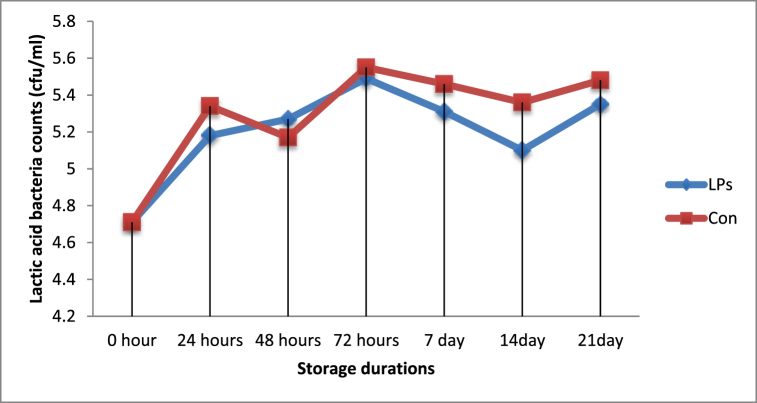
Lactic acid bacteria counts in yoghurt made from LPs activated and control milk samples. Abbreviations: LPs = Yoghurt made from activated milk and Con = Yoghurt made from inactivated milk.

This result pointed out that slightly lower counts were observed on yoghurt samples from LPs treated milk (Fig. 5). In addition, at some points of storage duration, yoghurt made from LPs treated milk had significantly lower counts as compared to yoghurt made from control milk. The current finding is in agreement with the finding of Seifu et al. [34], who found no significant differences in mold counts between the control and LPs activated goat milk cheese samples.

Coliforms and s*taphylococci* were not detected in all yoghurts from LPs activated and control milk samples throughout storage periods. The absence of s*taphylococci* and coliform counts in processed yoghurts can be attributed to the effect of pasteurization temperature, which eliminates these pathogenic microbes. The activation of the LP before pasteurization increases the efficiency of the thermal treatment, eliminating contamination with coliforms and thermo-resistant bacteria after treatment [22]. Magdoub et al. [32] also reported that, in yoghurt from LP system treated milk; coliforms were not detected throughout the storage period. Generally, the non-significant variation in microbial quality and lactic acid percentage at some points during storage might be due to the thermal treatment of milk coupled with the LPs [35,36].

### 3.4 Sensory properties of yoghurt

With respect to taste, there were no significant differences (*P > 0.05*) between the average scores given for yoghurt made from LPs treated and control. This was also true within each treatment. Concerning color, at 14 and 21days of storage, significant differences were observed (*P < 0.05*) and yoghurt from activated milk received higher scores. Until 14 days of storage, the yoghurt from LPs activated milk was the same, but in yoghurt from control only up to 7 days (Table 4).

**Table 4:** Sensory properties of yoghurt samples.^a^

Para	Group			Storage Duration (hours)						
				0	24	48	72	7day	14day	21day
Taste	LPs			4.75 ± 0.25	4.50 ± 0.29	4.50 ± 0.29	4.25 ± 0.25	4.50 ± 0.29	4.25 ± 0.25	3.75 ± 0.25
	Con			4.75 ± 0.25	4.50 ± 0.29	4.50 ± 0.29	4.00 ± 0.41	4.25 ± 0.25	3.75 ± 0.25	3.50 ± 0.29
P value				0.490	0.579	0.824	0.312	0.097	0.087	0.123
Color	LPs			4.50 ± 0.29^b^	4.25 ± 0.25^b^	4.50 ± 0.29^b^	4.50 ± 0.29^b^	4.50 ± 0.29^b^	4.25 ± 0.25^ba^	3.75 ± 0.25^a^
	Con			4.50 ± 0.29^b^	4.50 ± 0.29^b^	4.50 ± 0.29^b^	4.50 ± 0.29^b^	3.75 ± 0.25^ba^	3.25 ± 0.48^a^	3.25 ± 0.25^a^
P value				0.211	0.063	0.143	0.395	0.097	0.033	0.041
Flavor		LPs		4.50 ± 0.29^b^	4.25 ± 0.25^b^	4.00 ± 0.41^ba^	4.00 ± 0.41^ba^	3.75 ± 0.25^ba^	3.50 ± 0.29^a^	3.50 ± 0.29^a^
		Con		4.50 ± 0.29^d^	4.25 ± 0.25^dc^	3.75 ± 0.25^cba^	3.50 ± 0.29^ba^	3.50 ± 0.29^ba^	3.25 ± 0.48^ba^	3.00 ± 0.58^a^
P value				0.065	0.074	0.673	0.085	0.196	0.079	0.030
Smell		LPs		4.25 ± 0.25	4.50 ± 0.29	4.25 ± 0.25	4.25 ± 0.25	4.50 ± 0.29	4.00 ± 0.41	4.25 ± 0.25
		Con		4.00 ± 0.00^c^	4.00 ± 0.00^c^	4.25 ± 0.25^c^	4.25 ± 0.25^c^	3.75 ± 0.25^abc^	3.50 ± 0.29^b^	3.00 ± 0.41^a^
P value				0.067	0.096	0.807	0.051	0.099	0.211	0.044
Texture		Act	4.50 ± 0.25^bc^		4.25 ± 0.25^bc^	4.50 ± 0.25^bc^	4.75 ± 0.25^c^	4.00 ± 0.25^bac^	4.50 ± 0.29^bc^	3.75 ± 0.48^a^
		Con	4.50 ± 0.25^c^		4.50 ± 0.25^c^	4.50 ± 0.25^c^	4.50 ± 0.25^c^	3.75 ± 0.25^bc^	3.25 ± 0.48^c^	3.00 ± 0.41^a^
P value			0.362		0.094	0.174	0.561	0.086	0.069	0.098
Overall Acceptability		Act	4.50 ± 0.29^b^		4.50 ± 0.25^b^	4.50 ± 0.29^b^	4.50 ± 0.29^b^	4.50 ± 0.29^b^	4.25 ± 0.25^ab^	3.75 ± 0.25^a^
		Con	4.50 ± 0.29^b^		4.50 ± 0.29^b^	4.50 ± 0.29^b^	4.50 ± 0.29^b^	3.75 ± 0.25^ab^	3.25 ± 0.48^a^	3.25 ± 0. 48^a^
P value			0.314		0.065	0.099	0.072	0.115	0.002	0.027

Abbreviations: LPs, Lactoperoxidase system; NS, non-significant; Act, activated; Con, control.

^a^ Data presented are the means of triplicates with standard errors. Means in the same row with different superscripts are significantly different (P < 0.05).

**Fig. 7:**
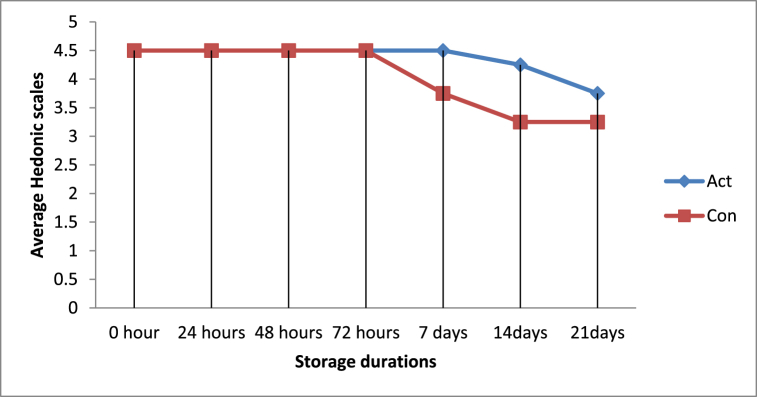
Overall acceptability of Yogurt samples made from LPs activated and control milk.

On day 21, significant differences (*P < 0.05*) were observed in flavor and smell between yoghurt made from activated and control milk, while no significant differences (*P > 0.05*) were observed due to treatments in the remaining storage days (Table 4). Particularly for average taste and smell scores, no significant differences (*P > 0.05*) were observed within yoghurt samples made from LPs treated milk across storage periods. However, significant differences (*P < 0.05*) were observed within the control group yoghurt. The texture of yoghurt from both groups didn't show significant variation (*P > 0.05*) across storage periods and within each treatment (Table 4). Generally, yoghurt made from LPs activated milk was preferred as compared to yoghurt made from control milk as the storage duration increased (Fig. 7). This study finding is in agreement with those of Masud et al. [24] who reported that yoghurts prepared from 10 ppm H_2_O_2_/NaSCN had high organoleptic quality as compared to those of the control. Other reports found that LPs does not induce adverse effects on sensory characteristics of raw milk and processed dairy products [21]. However, Ndambi et al. [23] indicated that yoghurt made from control samples was preferred to yoghurts made from LPs treated milk.

## 4 Conclusions

During incubation, samples from the control milk had a higher acidity percentage and a lower pH value than those from the activated samples at some incubation points. However, at the final stage of desired coagulum formation, significant differences were not observed. Lactoperoxidase system activation delayed lactic acid formation in yogurt, leading to a longer yogurt shelf life, during storage. LPs activation also improved the organoleptic quality of yogurt. Hence, using the LP system is a fairly relevant preservation system for raw cow milk prior to yoghurt making without a significant negative processing and storage quality influence. Besides, the application of the LPs could be crucial to integrating rural smallholder producers and milk processors. However, due consideration need to be given to heat deactivation of LPs before processing milk to yogurt into control the slower rates of fermentation observed in the current study. Further studies are recommended to assess LPs treated milk fitness for making other traditional and standard milk products and to identify the type of LAB in milk inhibited by LPs.

## Author contribution statement

Kedir Awol Assen: Conceived and designed the experiments; Performed the experiments; Analyzed and interpreted the data; Wrote the paper. Mestawet Taye Asfaw: Performed the experiments; Contributed reagents, materials and analysis tools or data; Wrote the paper. Binyam Kassa Engidasew: Analyzed and interpreted the data; Wrote the paper.

## Data availability statement

Data will be made available on request.

## Funding

The authors received no any specific and direct funding for this research work.

## Additional information

No additional information is available for this article.

## Declaration of competing interest

The authors declare that they have no competing financial interests or personal relationships that could have appeared to influence the work reported in this paper.
